# Evaluation of the Cognitive Function of Adults with Severe Hearing Loss Pre- and Post-Cochlear Implantation Using Verbal Fluency Testing

**DOI:** 10.3390/jcm12113792

**Published:** 2023-05-31

**Authors:** Manon Baranger, Valeria Manera, Chloé Sérignac, Alexandre Derreumaux, Elisa Cancian, Clair Vandersteen, Auriane Gros, Nicolas Guevara

**Affiliations:** 1 Département d’Orthophonie de Nice (DON), UFR Médecine, Université Côte d’Azur, 06107 Nice, France; valeria.manera@univ-cotedazur.fr (V.M.); auriane.gros@univ-cotedazur.fr (A.G.); 2 Laboratoire CobTeK, Université Côte d’Azur, 06100 Nice, France; derreumaux.a@chu-nice.fr; 3 Institut Universitaire de la Face et du Cou, Centre Hospitalier Universitaire, Université Côte d’Azur, 31 Avenue de Valombrose, 06100 Nice, France; chloemarieserignac@gmail.com (C.S.); elisa.cancian.ortho@gmail.com (E.C.); vandersteen.c@chu-nice.fr (C.V.); guevara.n@chu-nice.fr (N.G.); 4 Université Côte d’Azur, Centre Hospitalier Universitaire de Nice (University Hospital of Nice), Service Clinique Gériatrique du Cerveau et du Mouvement, Centre Mémoire Ressources et Recherche (Geriatric Brain and Movement Clinic, Memory Resources and Research Centre), 06100 Nice, France

**Keywords:** hearing loss, verbal fluency, cochlear implant, phonemic pathway, semantic pathway

## Abstract

Hearing loss is a major public health problem with significant evidence correlating it with cognitive performance. Verbal fluency tests are commonly used to assess lexical access. They provide a great deal of information about a subject’s cognitive function. The aim of our study was to evaluate phonemic and semantic lexical access abilities in adults with bilateral severe to profound hearing loss and then to re-evaluate a cohort after cochlear implantation. 103 adult subjects underwent phonemic and semantic fluency tests during a cochlear implant candidacy evaluation. Of the total 103 subjects, 43 subjects underwent the same tests at 3 months post-implantation. Our results showed superior performance in phonemic fluency compared to semantic fluency in subjects prior to implantation. Phonemic fluency was positively correlated with semantic fluency. Similarly, individuals with congenital deafness had better semantic lexical access than individuals with acquired deafness. Results at 3 months post-implantation showed an improvement in phonemic fluency. No correlation was found between the evolution of pre- and post-implant fluency and the auditory gain of the cochlear implant, and we found no significant difference between congenital and acquired deafness. Our study shows an improvement in global cognitive function after cochlear implantation without differentiation of the phonemic-semantic pathway.

## 1. Introduction

Approximately 25% of French adults are affected by hearing loss, of which 4% are at a disabling level [1]. A weak but significant correlation between hearing loss and cognitive performance has been reported [2]. Elderly people with hearing loss show an accelerated cognitive decline compared to their peers without hearing impairment [3,4,5,6] and thus a higher risk of dementia. In fact, according to the Lancet Commission, hearing loss is the largest potentially modifiable risk factor for dementia [7]. Several studies have shown promising results on the positive effects of hearing aid use on cognitive decline [8,9,10]. However, in cases of severe to profound hearing loss, the only reliable option for auditory rehabilitation is cochlear implantation [11].

At the cognitive level, cochlear implantation leads to improved performance in attention, memory [12,13] and inhibition [13]. Improvements in executive function tasks are greater in patients with lower baseline cognitive abilities [14].

Verbal fluency tests are regularly used to assess lexical access [15,16]. They provide information on memory storage capacity, the ability to retrieve stored information, the ability to organize thought and the strategies used to search for words [17]. These tests require that the participant produces as many words as possible from a specific category/condition in a limited period of time. Verbal fluency tests are performed under two main types of conditions: the phonemic condition (the subject is asked to produce words beginning with a certain given letter) and the semantic condition (the subject is asked to produce words from a certain given category). Verbal fluency tests reflect multiple high and low level cognitive abilities [16]. Both fluency tests require the integrity of lexical and semantic representations and of executive functions [15].

Semantic and phonemic fluencies depend on distinct neural systems. For phonemic verbal fluency, it can be seen with Functional Magnetic Resonance Imagining (fMRI) that the posterior regions of the left inferior frontal gyrus are more activated. On the other hand, for semantic fluency there is greater activation of the more anterior regions of the frontal and posterior regions of the temporal cortex [18,19].

Adults with severe to profound post-lingual hearing loss have a deterioration of phonological memory and its dorsal (fronto-parietal) pathway. The longer the subjects are exposed to this hearing loss, the more they use the ventral semantic (occipitotemporal) pathway to compensate for the lack of elementary phonological decomposition [20]. Phonological decomposition is an initial auditory step that enables secondary semantic analysis. Individuals who fail phonological decomposition lack linguistic analysis and correspondence between perceived and memorized phonology. This then limits the extraction of meaning from speech.

These difficulties with internal phonological representations and the degradation of auditory information worsen with the duration of auditory deprivation [21]. For post-lingual cochlear implant recipients, phonemic reconstruction is a more difficult cognitive task than semantic processing because of the degradation of auditory information delivered by the cochlear implant [22].

This study aims, firstly, to establish the phonemic and semantic lexical access abilities of adult subjects with bilateral severe to profound hearing loss and, secondly, to evaluate the impact of the cochlear implantation on phonemic and semantic lexical access.

## 2. Study 1

### 2.1. Materials and Methods

#### 2.1.1. Population

One hundred and three subjects participated in the present study (N = 103). The group consisted of 43 males and 55 females (mean age: 61.8 years; minimum 20 years; maximum 89 years). 15 subjects had primary school education, 36 subjects had secondary school education, and 49 had higher education. Data on education level was missing for 3 subjects. The origin of the hearing loss was variable: acquired hearing loss (*n* = 83), congenital hearing loss (*n* = 20). The etiologies are described in Table 1. The category “other” includes hearing loss from otitis associated with another context, head trauma, ototoxicity, toxic shock associated with progressive deafness, drug overdose, drug treatment, sepsis, as well as idiopathic deafness, and fragile X syndrome.

Subjects were recruited from a panel of patients with severe to profound hearing loss at the Institut Universitaire de la Face et du Cou de Nice during a pre-cochlear implant assessment. All subjects included had bilateral severe to profound hearing loss and were native French speakers. This study was approved by the Recherches Non Interventionnelles de l’Université Côte d’Azur (CERNI) AVIS number 2020-62. All participants signed an informed consent form before the start of the study.

#### 2.1.2. Materials and Procedure

We used Cardebat’s fluencies [23] to assess phonemic and semantic lexical access. The phonemic fluencies [P] and [R] and the semantic fluencies “animals” and “fruit” were used. Each subject took a phonemic fluency test and a semantic fluency test in a randomized fashion. For each fluency test, the participant had to produce as many words as possible within 2 min. The verbal fluency tests were given to the patients during the pre-cochlear implant assessment session.

#### 2.1.3. Statistical Analyses

The Z-score was calculated using Cardebat’s fluency calibration [23]. This calibration takes into account the type of fluency as well as the gender, age and education level of the participants. The Z-score is the standard deviation. The closer the Z-score is to 0, the closer it is to the norm. A negative Z-score shows below average performance, and a positive Z-score shows above average performance. The Shapiro-Wilk test was used to check the normal distribution of the data. Since the data is normally distributed, a paired-sample t-test was used to compare the Z-scores of phonemic and semantic fluency. In order to compare the phonemic and semantic fluency Z-scores between acquired and congenital deafness, we used an independent samples t-test and a Mann Whitney test according to the distribution of the data. The Pearson correlation was used to establish the correlation between the phonemic and semantic fluency Z-scores. Significant results are reported as *p* < 0.05 (*p* < 0.05 *, *p* < 0.01 **, *p* < 0.001 ***).

### 2.2. Results

Of the 103 participants, 2 individuals were below the pathology threshold (Z = −2) in phonemic fluency and 6 individuals in semantic fluency. Table 2 shows the results of the Z-scores for phonemic and semantic fluency.

**Figure 1:**
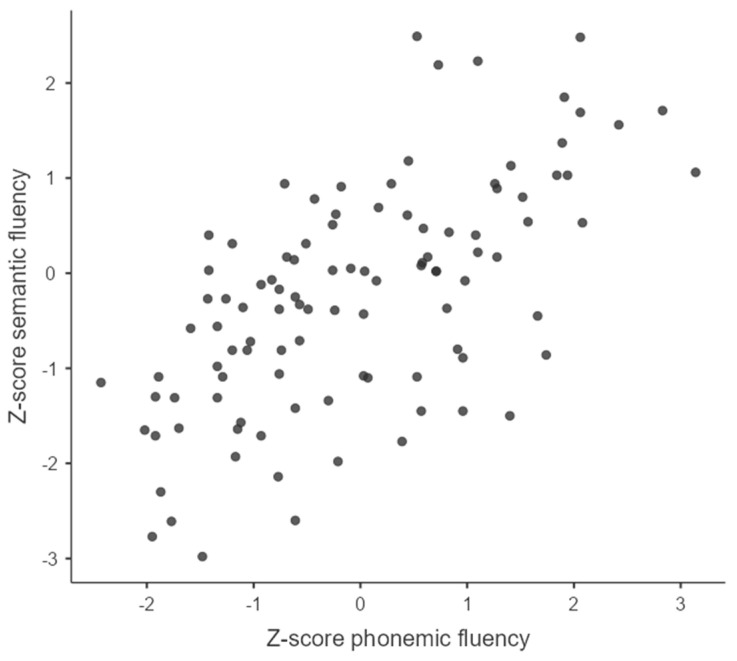
Distribution of Z-scores in the two fluency conditions.

The mean Z-score for phonemic fluency is higher than that for semantic fluency. The *p*-value shows that this result is significant (*p* = 0.043 *). In addition, there is a significant positive correlation between the phonemic fluency Z-score and the semantic fluency Z-score (*r* = 0.630, *p* < 0.001 ***). Figure 1 shows the distribution of phonemic and semantic fluency Z-scores.

The results of the phonemic and semantic fluency Z-scores according to the origin of the participants’ deafness (acquired deafness or congenital deafness) are described in Table 3.

The results show higher Z-scores in phonemic and semantic fluency for participants with congenital deafness. The *p*-value is significant for the Z-score in semantic fluency (*p* = 0.043 *). The *p*-value is not significant for the phonemic fluency Z-score.

## 3. Study 2

### 3.1. Materials and Methods

#### 3.1.1. Population

The subjects included in study 2 (N = 43) were from study 1. The group consisted of 19 males and 24 females (mean age: 55.7 years; minimum 20 years; maximum 85 years). 4 subjects (9%) had primary school education, 13 (30%) subjects had secondary school education and 25 (58%) had higher education. Data on the educational level of 1 (2%) subject was missing. The origin of the hearing loss was variable: acquired hearing loss (*n* = 31), congenital hearing loss (*n* = 12). The etiologies were diverse: 11 subjects had presbycusis, 5 subjects had congenital bilateral profound hearing loss, 3 subjects had otosclerosis-related hearing loss, 3 subjects had genetic hearing loss, 2 subjects had Pendred’s syndrome, 2 subjects had Meniere’s disease, 2 subjects had Meniere’s disease associated with chronic otitis, 2 subjects had hearing loss due to inner ear malformations, and 2 subjects had congenital deafness with abrupt worsening. 11 subjects had other etiologies: fragile X syndrome, autoimmune, idiopathic, meningitis, chronic ear infections, ototoxicity, sepsis.

At the end of the pre-implant assessment in Study 1, some participants had no indication for cochlear implantation. For other participants, data on verbal fluency at 3 months post-implantation were missing. These reasons explain the difference in participants between Study 1 and Study 2.

#### 3.1.2. Materials and Procedure

Different brands of cochlear implants were used: Medel (*n* = 15), Cochlear (*n* = 16), Advanced Bionics (*n* = 7) and Oticon Medical (*n* = 7).

Subjects were reviewed at a post-cochlear implant assessment session approximately 3 months after surgery. During this assessment, they were randomly retested for semantic and phonemic fluencies.

#### 3.1.3. Statistical Analyses

The Z-score was calculated following the fluency calibration of Cardebat [23]. This calibration takes into account the type of fluency as well as the gender, age and education level of the participants. Cochlear implant auditory gain was calculated as the difference between post-implant Pure Tone Audiometry (PTA) and pre-implant PTA. The Shapiro-Wilk test was used to check the normal distribution of the data. Since the data followed the normal distribution, a paired-sample t-test was used to compare the Z-score of pre- and post-implantation fluencies. In order to compare the differences in Z-scores of post-implantation and pre-cochlear implantation phonemic fluencies and post-implantation and pre-cochlear implantation semantic fluencies between acquired and congenital deafness, we used an independent samples t-test and a Mann Whitney test according to the distribution of the data. Pearson correlations were performed to analyse the relationship between differences in fluency scores and implant gain. Significant results are reported as *p* < 0.05 (*p* < 0.05 *, *p* < 0.01 **, *p* < 0.001 ***).

### 3.2. Results

Of the 43 subjects, one subject was below the pathological threshold (Z = −2) in phonemic fluency post cochlear implantation. For semantic fluency, 2 subjects were below the pathological threshold pre- and post-cochlear implantation. One participant went from a non-pathological to a pathological Z-score after implantation. One participant went from a pathological to a non-pathological Z-score after implantation.

Table 4 shows the results of the pre- and post-cochlear implantation Z-scores in the two fluency conditions (phonemic and semantic).

The results show a higher Z-score for post-implantation phonemic fluency than for pre-implantation phonemic fluency. The *p*-value shows that the result is significant (*p* = 0.024 *). In semantic fluency, the post-implantation Z-score is slightly higher than the pre-implantation Z-score, but this value is not significant (*p* = 0.863).

Table 5 describes the results of the differences in the post and pre cochlear implantation Z-scores according to the origin of the deafness (acquired deafness or congenital deafness).

The differences in phonemic fluency Z-scores post implant and pre implant show a gain for both groups. The mean is higher in the group of subjects with acquired hearing loss than in the group with congenital hearing loss.

The difference in semantic fluency Z-scores post implant and pre implant shows a small gain for the group of subjects with an acquired hearing loss. For subjects with congenital hearing loss the difference in semantic fluency Z-scores post implantation and pre cochlear implantation shows a small loss. No significant difference was found between the two groups in the post-implantation condition in either fluency (*p* = 0.751 and *p* = 0.692).

**Figure 2:**
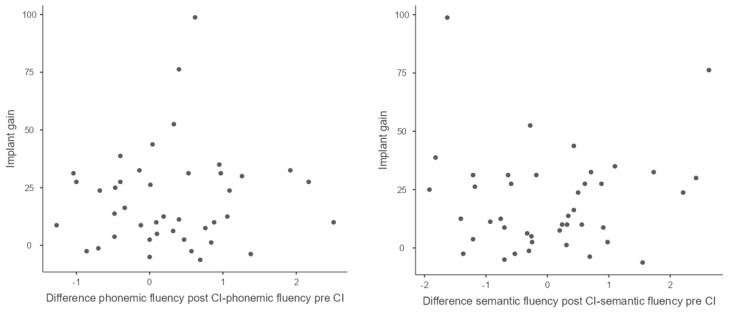
Distribution of post- and pre-implantation Z-score differences and implant gain.

Figure 2 shows the distribution of the differences in Z-scores (in phonemic and semantic fluency) post and pre-implant and implant gain.

**Figure 3:**
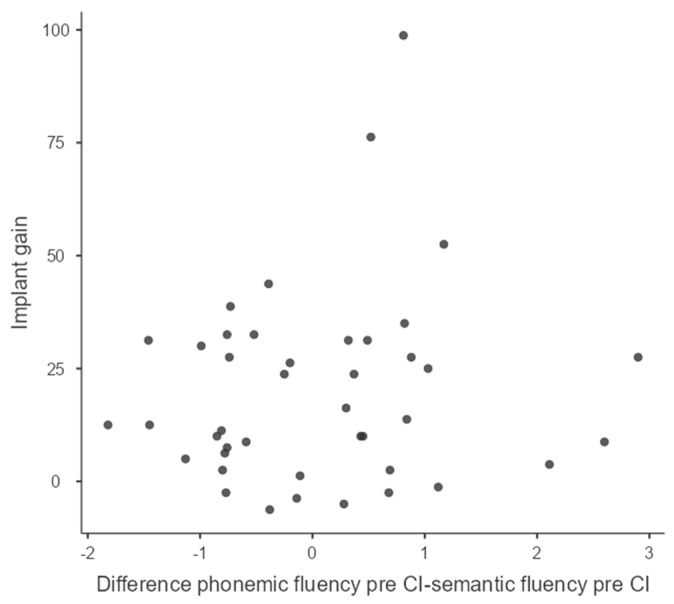
Distribution of pre-implant phonemic and semantic Z-score differences and implant gain.

Figure 3 shows the distribution of pre-implantation phonemic and semantic Z-score differences and implant gain.

The results show no correlation between pre-implantation phonemic and semantic Z-score differences and implant gain (*r* = 0.119, *p* > 0.05).

## 4. Discussion

The first aim of this study was to establish the lexical access abilities of adult subjects with bilateral severe to profound hearing loss. Study 1 showed superior performance on phonemic fluency compared to semantic fluency in the subjects included. This result is in contrast with the Santos study which showed superior performance on semantic fluency in adults with hearing impairment [24]. However, our study and Santos’ study were not conducted in the same language (French vs. Brazilian Portuguese). The time allotted to measure the fluencies was also different (2 min vs. 1 min). It has been shown that semantic fluency is an indicator of a deficit in executive function [25]. Similarly, it is known that hearing impairment affects executive functions [26]. The results of this study could, therefore, demonstrate an executive function deficit more pronounced than the phonological deficit in adults with severe to profound hearing loss. Furthermore, the maintenance of this dorsal phonemic pathway in subjects with post-lingual hearing loss predicts a favourable outcome with a cochlear implant [21].

A positive correlation was observed between the phonemic and semantic Z-scores. Therefore, there is a link between success in phonemic and semantic fluencies, even though phonemic and semantic fluencies involve separate distinct systems. Semantic fluency involves the inferior longitudinal fasciculus, the unciform fasciculus, the temporal part of the inferior fronto-occipital fasciculus and the superior temporal gyrus. Phonemic fluency involves the ascending frontal tract, the frontal part of the inferior fronto-occipital bundle and the superior frontal gyrus [27]. The results of our study suggest a close link between these two pathways and that they can work together.

People with congenital hearing loss have better semantic lexical access than people with acquired hearing loss. This can be explained by the fact that people with congenital hearing loss have insufficient phonological decomposition dating to the prelingual period. Therefore, they would preferentially use the ventral (occipito-temporal) semantic pathway [20].

Secondly, we assessed the impact of cochlear implantation on phonemic and semantic lexical access. Study 2 showed a positive impact of the cochlear implant on phonemic fluency as early as 3 months post implant. Thus, cochlear implantation allowed for better phonological representation and better access to the dorsal pathway. This benefit of the cochlear implant appears relatively early after surgery. These results are important because most studies have highlighted cognitive changes at six months or one year but not so early. Indeed, since ten years the clinical research on the cognitive improvement by cochlear implantation has become increasingly recognized. Especially, studies have showed improvements in different cognitive functions as processing speed, cognitive flexibility and working memory [28,29,30]. These studies have conducted prospective longitudinal studies as early as 6 months after cochlear implantation but never as early as 3 months. So, our results suggest that verbal fluency improvement start since 3 months after implantation.

Study 2 showed no significant difference in pre- and post-cochlear implantation fluency between groups based on the origin of the hearing loss. The contribution of the cochlear implant on phonemic and semantic fluency is as effective for an acquired hearing loss as for a congenital one at 3 months post-implant. No correlation was found between differences in post- and pre-implant fluency and implant auditory gain. At 3 months post-implantation, the benefit of the cochlear implant is not specific to the phonemic and semantic pathways. The distribution between the pathways is not determined.

Finally, the improvement in participants verbal fluency, even elderly, and this very quickly, adds proof of the interest of cochlear implantation for cognitive stimulation and the prevention of cognitive decline as suggested by other studies [31].

It would be interesting to continue this study with a longer post cochlear implantation observation time to see if a correlation could be obtained between implant gain and the difference in phonemic and semantic fluency. It might also be interesting to analyse clustering (retrieval of words by phonemic or semantic subcategory) and switching (moving from one subcategory to another) in verbal fluency tasks in people with hearing impairment.

### Study Limitations

A major limitation of our study is the short post-implantation interval. We observed verbal fluency only 3 months after implantation whereas most studies analyse cognition 1 year after implantation. Indeed, activation of the auditory associative cortex continues to increase even years after cochlear implantation for stimuli containing speech [32]. Another limitation of this study is the calibration used. The Cardebat fluencies are calibrated for age, gender and education level. The age norms range from 30 to 85 years. However, in this study, participants under 30 and over 85 years of age were included. The calibration used for these participants was the one closest in age respecting gender and education.

## 5. Conclusions

Our study showed higher phonemic fluency than semantic fluency, in contrast to previous studies. At 3 months after surgery, we observed a benefit of the cochlear implant in adults without differentiation between the phonemic and semantic pathways and regardless of the origin of the hearing loss. This study could be continued over a longer period of observation post cochlear implantation. It might be relevant to compare the impairment of phonemic and semantic pathways with that of executive functions in adults with hearing impairment.

## Figures and Tables

**Table 1:** Etiologies of hearing loss in the population ranked by frequency.

Etiologies	N
Presbycusis	15
Meniere’s disease	10
Otosclerosis	8
Congenital bilateral profound deafness	5
Meningitis	3
Malformation	3
Chronic otitis	3
Hereditary	3
Genetic	3
Meniere’s disease with chronic otitis	2
Infection	2
Congenital deafness with sudden aggravation	2
Autoimmune	2
Pendred’s syndrome associated with malformation	2
Other	10
Unknown data	30

**Table 2:** Phonemic and semantic fluency results by Z-score.

	Z-Score Phonemic Fluency (N = 103)	Z-Score Semantic Fluency (N = 103)
Mean	−0.0446	−0.256
Standard deviation	1.25	1.18
Minimum	−2.43	−2.98
Maximum	3.14	2.49
*p*-value	0.043 *	

**Table 3:** Phonemic and semantic fluency Z-scores according to the origin of the deafness.

	Acquired Deafness (N = 81)/Congenital Deafness (N = 22)	Z-Score Phonemic Fluency	Z-Score Semantic Fluency
Mean	Acquired	−0.109	−0.379
	Congenital	0.191	0.194
Standard deviation	Acquired	1.26	1.21
	Congenital	1.22	0.974
Minimum	Acquired	−2.43	−2.98
	Congenital	−1.43	−1.50
Maximum	Acquired	3.14	2.49
	Congenital	2.06	2.48
*p-*value	/	0.330	0.043 *

**Table 4:** Phonemic and semantic fluency Z-score results pre and post cochlear implantation.

	Z-Score Phonemic Fluency Pre-CI	Z-Score Phonemic Fluency Post CI	Z-Score Semantic Fluency Pre-CI	Z-Score Semantic Fluency Post CI
Mean	0.00116	0.302	−0.0635	−0.0342
Standard deviation	1.30	1.15	1.16	1.41
Minimum	−1.95	−2.25	−2.77	−2.27
Maximum	2.83	2.46	2.23	4.00
*p-*value	0.024 *		0.863	

**Table 5:** Results of Z-score differences in post- and pre-cochlear implant phonemic fluency and post- and pre-cochlear implant semantic fluency between acquired and congenital deafness.

	Acquired Deafness (N = 29)/Congenital Deafness (N = 14)	Z-Score Difference Phonemic Fluency Post CI- Z-Score Phonemic Fluency Pre-CI	Z-Score Difference Semantic Fluency Post CI- Z-Score Semantic Fluency Pre-CI
Mean	Acquired	0.329	0.0766
	Congenital	0.241	−0.0686
Standard deviance	Acquired	0.843	0.971
	Congenital	0.873	1.38
Minimum	Acquired	−1.27	−1.92
	Congenital	−1.04	−1.82
Maximum	Acquired	2.51	2.63
	Congenital	2.17	2.42
*p-*value	/	0.751	0.692

## Data Availability

Data will be provided upon the request to researchers.
